# Extracting Neural Oscillation Signatures of Laser-Induced Nociception in Pain-Related Regions in Rats

**DOI:** 10.3389/fncir.2017.00071

**Published:** 2017-10-09

**Authors:** Xuezhu Li, Zifang Zhao, Jun Ma, Shuang Cui, Ming Yi, Huailian Guo, You Wan

**Affiliations:** ^1^Neuroscience Research Institute, Peking University, Beijing, China; ^2^Department of Neurology, People’s Hospital, Peking University, Beijing, China; ^3^Department of Neurobiology, School of Basic Medical Sciences, Peking University, Beijing, China; ^4^Key for Neuroscience, Ministry of Education/National Committee of Health and Family Planning of China, Peking University, Beijing, China

**Keywords:** acute pain, electroencephalogram, neural oscillation, machine learning, pain network

## Abstract

Previous studies have shown that multiple brain regions are involved in pain perception and pain-related neural processes by forming a functionally connected pain network. It is still unclear how these pain-related brain areas actively work together to generate the experience of pain. To get a better insight into the pain network, we implanted electrodes in four pain-related areas of rats including the anterior cingulate cortex (ACC), orbitofrontal cortex (OFC), primary somatosensory cortex (S1) and periaqueductal gray (PAG). We analyzed the pattern of local field potential (LFP) oscillations under noxious laser stimulations and innoxious laser stimulations. A high-dimensional feature matrix was built based on the LFP characters for both experimental conditions. Generalized linear models (GLMs) were trained to classify recorded LFPs under noxious vs. innoxious condition. We found a general power decrease in α and β bands and power increase in γ band in the recorded areas under noxious condition. After noxious laser stimulation, there was a consistent change in LFP power and correlation in all four brain areas among all 13 rats. With GLM classifiers, noxious laser trials were distinguished from innoxious laser trials with high accuracy (86%) using high-dimensional LFP features. This work provides a basis for further research to examine which aspects (e.g., sensory, motor or affective processes) of noxious stimulation should drive distinct neural activity across the pain network.

## Introduction

Pain experience is a complex neural process that involves sensory, emotional and cognitive systems (Melzack and Casey, 1968). Functional imaging studies have shown that a “pain matrix” consisting of multiple functionally connected brain areas is formed during pain (Tracey and Johns, 2010). When an external noxious stimuli are applied to a human body, multiple pathways are activated at the same time. Somatosensory information is processed in somatic sensation-related areas including thalamus (LeBlanc et al., 2014), primary somatosensory cortex (S1; Vierck et al., 2013; LeBlanc et al., 2016) and secondary somatosensory cortex (S2; Timmermann et al., 2001; Hsiao et al., 2008). Negative emotion arises from emotion-related areas like anterior cingulate cortex (ACC; Rainville et al., 1997; Hauck et al., 2015) while decision and cognitive components are formed in orbitofrontal cortex (OFC; Ochsner et al., 2006) and insula (Bastuji et al., 2016). Pain experience is also remembered. Memory-related areas like hippocampus and medial prefrontal cortex actively become part of the pain network (Buzsáki, 1989; Buzsáki and Moser, 2013). The final port of top-down regulation of pain is periaqueductal gray (PAG; Green et al., 2009), which inhibits nociceptive input by inhibiting nociception afferent fibers (Heinricher et al., 2009).

Recent studies have suggested that a “pain center” does not exist, but instead pain-related areas form a “pain matrix” together (Ploner et al., 2016). Any part of this pain matrix by itself is not pain-specific. But the network exhibits a unique pattern during pain as a whole.

For the sparsely distributed brain areas of the pain matrix to work together, they have to be functionally connected. Neural oscillation provides a transient time window for different neural assemblies to work together (Buzsáki and Draguhn, 2004). Thus, we hypothesize that by learning the dynamic patterns of oscillations in different brain areas, we could extract the neural oscillation signature to get a deeper insight into the functional dynamics of pain.

To investigate the neural oscillations among several brain areas, extracellular electrophysiological recording is now the best technique (Buzsáki et al., 2015). By implanting electrodes directly to the regions of interests, local field potentials (LFPs) can be recorded (Buzsáki et al., 2012) and used to reveal information about local oscillatory neural activities. An LFP signal can be further broken down into different frequency bands of oscillation (Buzsáki and Draguhn, 2004). Each frequency band contains both amplitude and phase information. Correlations could be found among different frequency bands of oscillation and between different brain areas (Buzsáki and Schomburg, 2015). Neural oscillations have been repeatedly reported to be correlated to pain. Under noxious stimulation, decreased power of α and β bands, increased power of γ band (Ploner et al., 2006; Hauck et al., 2015) and increased θ—γ coherence have been reported (Wang et al., 2011).

When LFP signals are used to study the dynamics of a complex neural network, the high dimensionality of LFP feature space brings a big challenge to data processing. The power changes in pain-related areas and the coherence changes in pain network exhibit a high dimensional feature space. Thus it requires dimension reduction techniques for neural computation. Dimension reduction and feature extraction techniques are widely used in analysis of neural data (Shen and Meyer, 2006; Wang et al., 2007; Pereira et al., 2009). Machine learning classifiers can be used to extract useful information hidden in the high dimensional data space (Haynes and Rees, 2006). Similarly, neural oscillation patterns within the pain network could be extracted using a machine learning based algorithm.

In the present study, we implanted microelectrodes in the ACC, OFC, S1 and PAG of rats. We monitored the neural oscillation changes under noxious laser stimulation and innoxious laser stimulation. Generalized linear model (GLM) classifiers were trained to classify noxious and innoxious conditions with high accuracy. We found that noxious laser stimulation trials were distinguished from the innoxious laser stimulation trials with high accuracy (89%) using high-dimensional LFP features.

## Materials and Methods

### Animal and Surgery

Experiments were performed on 13 adult male Sprague-Dawley rats provided by the Department of Laboratory Animal Science of Peking University Health Science Center. All experiments were carried on following the guidelines of the Institutional Animal Care and Use Committee of Peking University.

Rats weighting 280–300 g were housed individually under 12-h dark-light cycle with free access to food and water. Surgeries were performed when their body weight reached 330–350 g. During surgery, the rat was anesthetized with 1% sodium pentobarbital (0.5 ml/kg). Supplementary doses of less than 1/3 of the original dose were added when necessary to maintain anesthesia. A pinch test was done to the hindpaw of the rat to check the depth of anesthesia. The head of the rat was fixed to the Kopf stereotaxic apparatus (David Kopf Instruments, Tujunga, CA, USA) with ear-bars. Then the skull was exposed. Coordinates of regions of interests were determined according to the atlas of rat brain coordinates (Paxinos and Watson, 2009) as follows (in mm): ACC, anterior/posterior (A/P) + 2.3, medial/lateral (M/L) + 0.7, dorsal/ventral (D/V) −2.2; OFC, A/P +3.7, M/L +2.7, D/V −5.3; S1: A/P −1.1, M/L −2.6, D/V −2.0; PAG: A/P −7.6, M/L +0.8, D/V −6.0. Six stainless screws were tightened onto the skull without piercing through the dura. Screws acted as anchors for electrodes stabilization, while two of them were used as ground and reference, respectively. Small craniotomies were performed at corresponding coordinates. Dura was removed carefully to expose the brain tissue. Micro-wire electrode arrays (Blackrock Microsystems Ltd., manufactured in China) were lowered into target regions separately with a low insertion speed (1 mm/min) and then bounded to the screws using dental cement. Finally, all arrays were fixed to the cranium using dental cement. After the surgery, each rat was allowed to recover for 1 week in its individual cage with free access to food and water.

### Laser Stimulation

The rat was allowed to move freely in a transparent plastic chamber with video recording devices. The chamber floor was a grid plate with stainless steel bars of 2 mm in diameter and 8 mm in between. Headstages were connected to the electrode connectors on the head of the rat for electrophysiology recording.

When the rat was awake and quietly lying down, laser stimulation was applied to its left hindpaw. A laser beam was emitted from the guide arm of an ultra-pulse carbon dioxide laser therapeutic machine. The tip of the guide arm kept a distance of 2 cm to the plantar surface of the paw. Focus of the laser beam was changed a little bit from session to session to avoid any possible tissue damage. A synchronized video was recorded during electrophysiology recording. The power of noxious laser stimulation was set to a range from 8 Watts to 12 Watts with an emission time of 30 ms. As self-control, innoxious laser stimulation of 4 Watts was applied to the animal while other parameters were kept unchanged. Nociceptive behavior was identified by observation of immediate paw withdrawal after stimulation. Noxious stimulation power was adjusted individually according to the occurrence of nociceptive behavior. Noxious laser stimulation trials without paw-lifting were excluded in the further analysis. Each recording session contained 20 control trials and 20 noxious trials with an inter-stimulus interval of no less than 60 s to avoid hyperalgesia. The rat was allowed to rest for 2 days between recording sessions. Four recording sessions were conducted for each rat.

### Electrophysiology Recording

The multi-channel recording system was manufactured by Blackrock Microsystems Limited (Salt Lake City, UT, USA). Electrophysiological data were recorded from the implanted micro-wire electrode arrays. Four pre-amplification headstages were used to record from 32 electrodes. The analog signals were filtered by a band-pass filter set between 0.3 Hz and 7500 Hz. Then the signals were digitized by the neural signal processor. The LFPs were recorded at 1 kHz/s sampling rate. The single unit spikes were manually sorted online by Central, a computer software provided by Cerebus (Blackrock Microsystems Ltd., Salt Lake City, UT, USA).

### Histology

To verify the placement of the implanted electrodes and biocompatibility of the system, histology was performed to all of the implanted animals. The rat was deeply anesthetized with urethane injection. One microampere pulse was delivered to each recording electrode to create a marker at the recording site. Then the animal was perfused via heart with 0.9% saline followed by 4% paraformaldehyde in 0.12 M sodium phosphate buffer (pH = 7.4). Three-hundred microliter fixative was used per 100 g of body weight. After perfusion, the brain was removed from the skull, and post-fixed in the same fixative at 4°C for more than 24 h before moved to 10× PBS overnight. The fixed brain was cut into 50-μm thick slices. Histology results are shown in Supplementary Figure S1.

### Data Pre-Processing

To remove movement artifacts and other correlated noise, independent component analysis (ICA, EEGLAB toolbox) was applied to all recorded channels first. ICA algorithm was used to isolate sources with different spatial distribution while conserve their temporal information. Decomposed independent components (ICs) were inspected by both waveforms and weight distribution across recording channels manually. ICs with slow and large fluctuations temporally correlated to animal movement were treated as movement artifacts and thus removed from further analysis. ICs with uniformly distribution across multiple areas were considered as volume conduction effect and thus also eliminated from data (Supplementary Figure S2). We used an open-source neural data visualizer to visualize waveforms of each laser-triggered event (Hazan et al., 2006). Events with large movement artifacts were ruled out from further analysis. After source identification process, ICs were converted back to original waveform.

Out of the eight channels from the same recording site, one channel was selected to represent the local activity of the region of interests. The channel with highest gamma band activity was selected (Sirota et al., 2008). By the end of the data pre-processing, four de-noised LFP channels were prepared for further analysis.

### Feature Extraction

Oscillation characterizations were extracted in following steps.

1. For each laser trial, LFPs were filtered to δ (0–4 Hz), θ (4–8 Hz), α (8–12 Hz), β (13–30 Hz), γ (30–80 Hz) and ε (80–120 Hz) bands with a non-causal IIR filter (filtfilt in MatLab).
2. The analytical signals were obtained by applying Hilbert transform to the filtered data. For a temporal sequence *a*(*t*), the corresponding Hilbert transform was calculated by following equation (*Hilbert* in MatLab):
  (1) $${\text{H}}_{a}(t)=\frac{1}{\pi }P.V.\;\int_{-\infty }^{+\infty }\frac{a(t)}{t-\tau }\text{dτ}$$
  Here, P.V. indicates that the integral is taken in the sense of Cauchy principal values.
3. LFP power was calculated by two steps. The first step was extracting signal envelop by taking the absolute values of the previous calculated analytical signals. The second step was to calculate averaged LFP powers of pre-stimulation window and post-stimulation window by taking median values over power envelop in the time windows of −2 s to 0 s and 0.5 s to 2.5 s. We added an offset of 0.5 s to the post-stimulus calculation window to avoid the effect of evoked-potentials.
4. Amplitude envelop correlation (AEC) is a straightforward coupling detection algorithm for incoherent brain signals (Bruns et al., 2000). For a pair of filtered signal segments *a*(*t*) and *b*(*t*), their corresponding Hilbert transforms are H_a_(*t*) and H_b_(*t*). AEC between *a*(*t*) and *b*(*t*) is defined by the following equation:
  (2) $$\text{AEC}=\;\frac{\sum_{t\;=\;1}^{n}\left(\left|{\text{H}}_{a}(t)|-|\bar{H_{a}(t)}\right|\right)\left(\left|H_{b}(t)|-|\bar{H_{b}(t)}\right|\right)}{\sqrt{\sum_{t\;=\;1}^{n}{\left(\left|H_{a}(t)|-|\bar{H_{a}(t)}\right|\right)}^{2}}\;\sqrt{\sum_{t\;=\;1}^{n}{\left(\left|H_{b}(t)|-|\bar{H_{b}(t)}\right|\right)}^{2}}}$$
5. To visualize the time-frequency feature of the raw data, Gabor transform (WaveLab toolbox^1^) was applied to the raw data around each event time.

In the final stage, data from all animals (*N* = 13) and all recording sessions were collected and concatenated together (*N* = 1622). Spatial variation of electrode, electrode impedance and animal difference resided in the data were non-nociception related variations and needed to be eliminated. A standardization process was applied to all recording sessions to rule out such variations. For each recording session, all pre-stimulation features were collected to calculate the mean and standard deviations. Then for the specific recording session, all features were z-scored by previously calculated mean and standard deviations. After this step, data from all sessions were concatenated together to build the final feature matrix. Feature matrix is an M-by-N matrix which contains M features and N trials. M features (*M* = 336) contains the average power of each oscillation band (*M*_1_ = 4 × 6) in each brain region and the AEC between each pair of oscillations (*M*_2_ = 312). After pre-processing, 1622 valid trials of data were collected in the feature matrix (control laser trials = 708, noxious laser trials = 914).

### Data Visualization with t-Distributed Stochastic Neighbor Embedding

Extracted features were concatenated to a trial by the feature matrix. Then the data were sent to t-Distributed Stochastic Neighbor Embedding (t-SNE) toolbox (MatLab implementation downloaded in t-SNE website) to get a 2D representation.

### Training Generalized Linear Model Classifiers

Generalized linear regression function in the MatLab was used to perform all GLM training and prediction. A linear model was adopted and a binomial link function was used to fit innoxious trials and noxious trials. In our experiment, we trained the models in two steps. The initial model was trained by data from 12 rats (*N* = 2972) with all LFP features to check the contribution of each LFP feature to the classification of noxious and innoxious laser trials. Data from a separate rat was used as a prediction dataset (*N* = 272). A 10-fold cross-validation evaluation was used to test the models. Data was cut into 10 randomized subsets and each subset was using as a testing set while others were used to train the models. After each training process, prediction set was sent to the model to test the prediction accuracy. After acquiring the initial model, coefficients of each feature dimension were calculated and sorted by their absolute value. Then new GLMs trained with a dimension-reduced feature matrix with an increasing number of selected features. In the final test, the best GLM was applied to all data segments in the whole recording dataset (92 dimensions were used). GLMs were trained by a strict time-window around stimulus onset-time. But in the final test, we performed a window-by-window evaluation in which most of the data were totally new to the models while the rest time windows around stimulus were not aligned to the windows used in the training phase. This test was intended to check the generalization ability of our model because the model has never seen most of the data between laser onsets.

### Statistics

Features were normalized by computing corresponding z-scores in a session-by-session manner. In each recording session, a pre-stimulation window (−1 s to 0 s) and a post-stimulation window (0.5 s to 1.5 s) were selected for each laser stimulation trial. All pre-stimulation data were collected to calculate a median value $\bar{x}$ and a standard deviation σ. Then data from both time windows were normalized in the following equation:

$$z=\frac{x-\bar{x}}{\sigma }$$

Two-sample *t*-tests were then performed to each normalized feature to check the statistical significance. Data for t-SNE training and GLMs were also based on this normalization process.

## Results

### Noxious Laser Stimulation-Evoked LFP Changes

**Figure 1:**
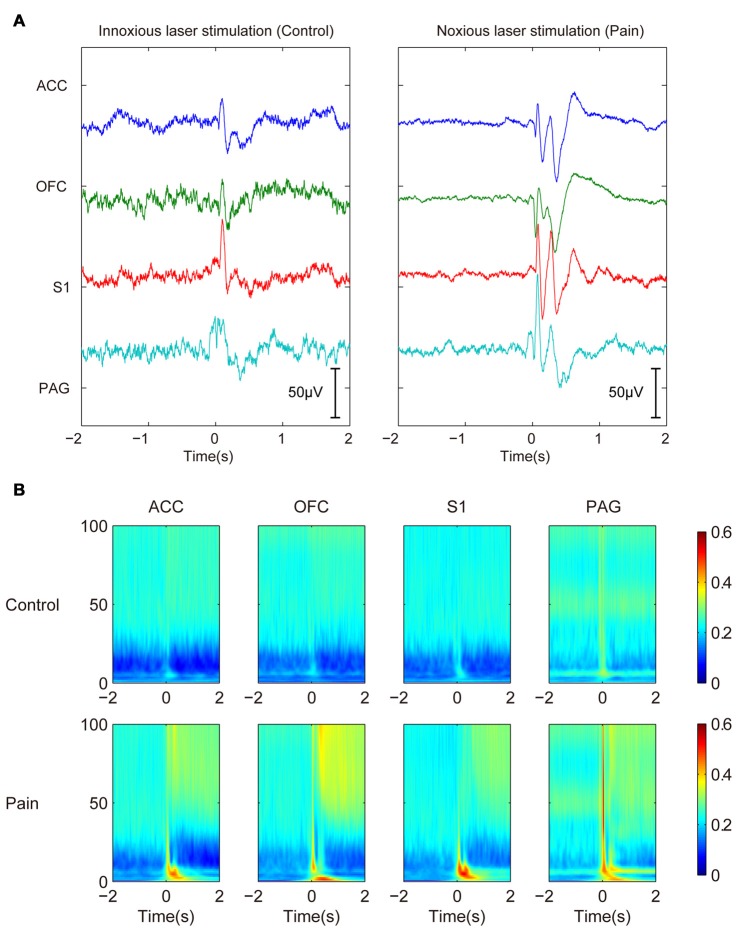
Trial-averaged local field potentials (LFPs) show persistent power changes in γ band and inhibition in β band after noxious stimulation. **(A)** Trial-averaged raw field potentials. Left panel shows LEPs of control innoxious laser stimulation. Right panel shows LEP of noxious stimulation. LEP lasts about 500 ms. **(B)** Gabor transform of LFPs. Control group is shown in the upper panel, and Pain group is shown in the bottom panel. Persistent power changes are observed in γ band and inhibition in β band after noxious stimulation.

After the removal of movement artifacts, volume conduction and cable-related noises by ICA decomposition, we examined the laser-induced LFP changes. A prominent transient evoked potential could be observed shortly after the stimulation onset (0–500 ms). Because the evoked potential was more related to the presence of stimulation and lasted for a relatively fixed duration, we focused more on the neural oscillations that followed. Laser evoked potential showed different LFP responses under innoxious and noxious conditions (Figure 1A). The noxious group (with noxious laser stimulation) had a more sustained response with clearer P1, N1, P2, N2 components than the innoxious group (with innoxious laser stimulation). However, in our present study, evoked potentials are not our main focus. Change of oscillatory LFP activities was the focus in this work. We used a gabor transform showed the averaged LFP changes in time-frequency domain (Figure 1B). The most distinct changes were suppression in α and β bands and a general increase in δ and γ bands after the noxious stimulation. In the following analysis, we selected the time window from 500 ms to 1500 ms after the stimulation onset and 0 ms to 1000 ms before the stimulation onset as post-stimulus and pre-stimulus time window, respectively. We added the 500-ms delay in the post-stimulus window to avoid the effect of evoked potentials.

**Figure 2:**
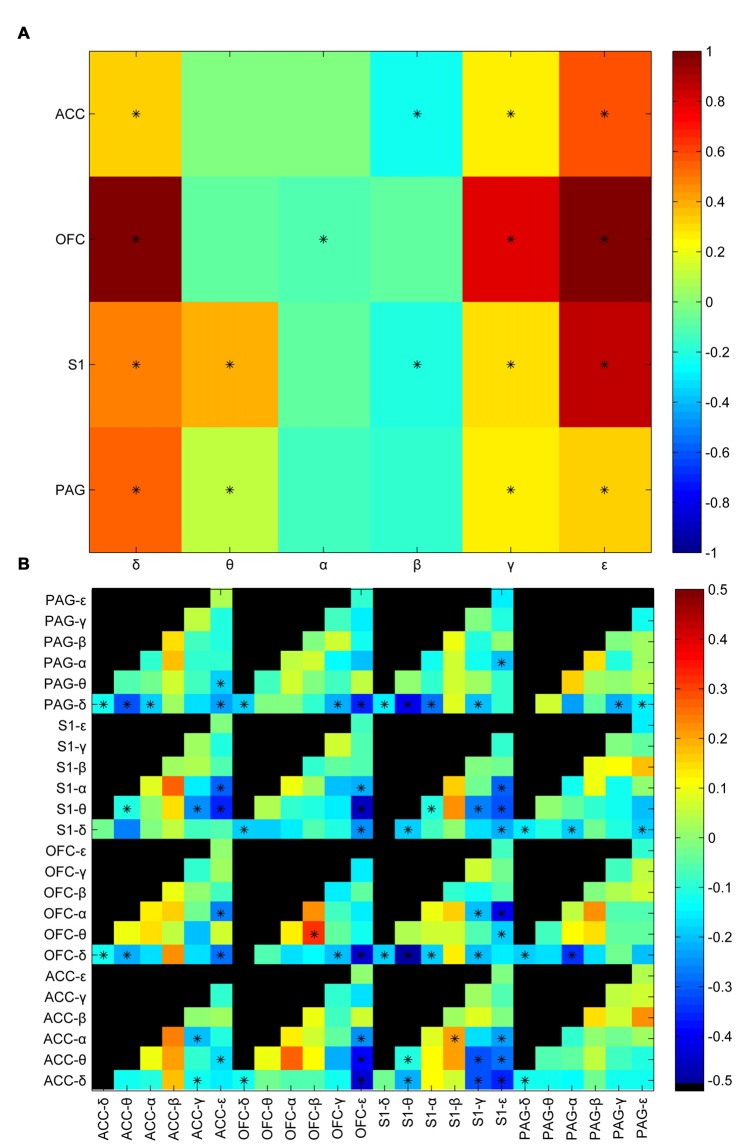
Laser-induced oscillatory changes between noxious and innoxious groups. **(A)** Averaged power difference in the post stimulus time window within brain areas (anterior cingulate cortex (ACC), orbitofrontal cortex (OFC), primary somatosensory cortex (S1) or periaqueductal gray (PAG)) between noxious group and innoxious group. Different colors indicate the power difference (Blue to Red correspond to −0.5 to 0.5). Prominent power increase is observed in δ, γ and ε bands in all recorded areas. Power decreases in α and β bands in ACC, S1 and PAG. Asterisks indicate statistical significance (*p < 0.05, Holm-Bonferroni test). **(B)** Averaged difference of amplitude envelop correlation (AEC) in the post stimulus time window among different brain areas between noxious group and innoxious group. Normalized mean differences of AEC are indicated by different colors (Blue to Red correspond to −0.5 to 0.5, Black indicates no value for the corresponding combination). Asterisks indicate statistical significance (*p < 0.05, Holm-Bonferroni test).

LFP signals in the corresponding time windows were further filtered into six oscillation bands (in Hz) including δ (0–4), θ (4–8), α (8–12), β (12–30), γ (30–80), ε (80–120). Power envelops were extracted by Hilbert transform. Average power was calculated by taking average of the power envelop of the analyzing window. Then a standardization process was performed to maintain the relative difference between the post-stimulus window and pre-stimulus window. Figure 2A shows the statistic results of the post-stimulus power change between innoxious group and noxious group. There was a statistically significant difference between innoxious group and noxious group among most power bands. δ, γ, ε band power were generally increased, meanwhile α, β band power were generally decreased. Correlations between power envelops were also checked (Figure 2B). Among all checked envelop pairs (*n* = 312), about one third of the parameter sets showed a statistically significant difference between innoxious group and noxious group (*n*_*p*<0.05_ = 80). These results indicated that noxious laser stimulation induced a broad impact on LFP activities.

In order to get a more intuitive visualization of the hidden data structure residing in the high dimension electrophysiological feature space, a dimension reduction technique called t-SNE was adopted. High dimensional data was casted to a 2-D space with maximal information entropy preserved. By doing so, we were able to visualize the similarity between different datasets and refine our data processing. Supplementary Figure S3 shows the t-SNE results. Noxious post-stimulation trials gathered together while the rest (innoxious pre-stimulation, noxious pre-stimulation and innoxious post-stimulation) formed another cluster in the 2-D t-SNE space.

### Electrophysiological Indicators Classifying Noxious from Innoxious Trials with High Accuracy

**Figure 3:**
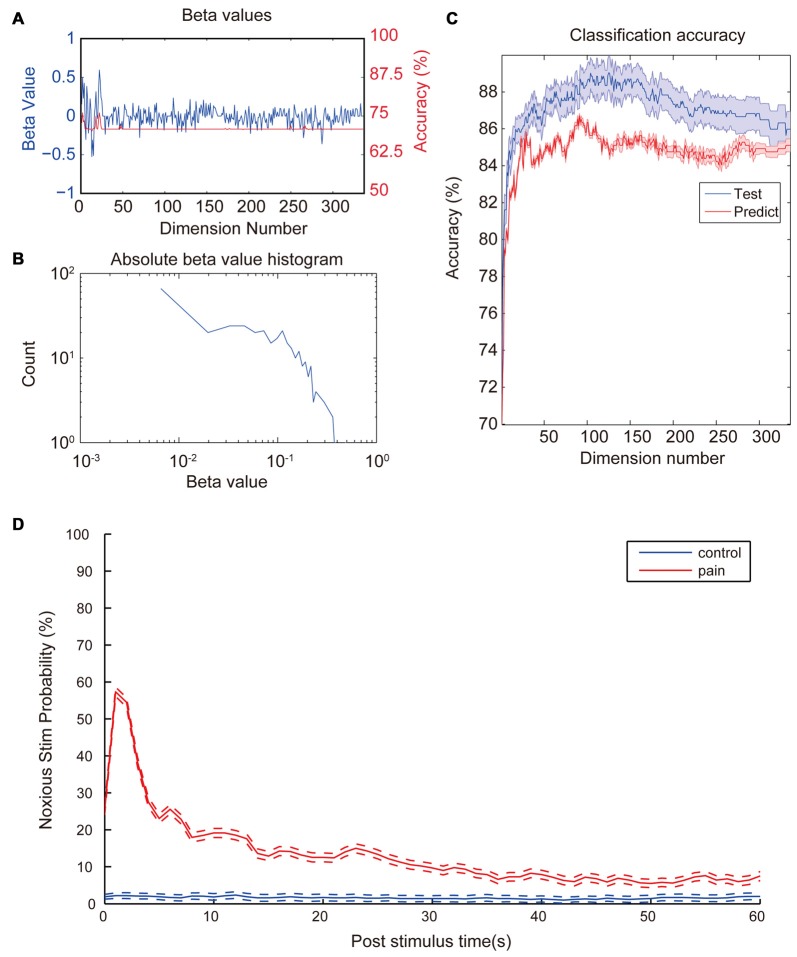
Coefficient values and prediction rate of generalized linear models (GLMs). **(A)** β-values of the initial GLM classifier which trained with the whole dataset (Blue). Classification accuracy of GLMs trained with the corresponding LFP feature in the test set (Red). **(B)** Histogram of absolute β-values. Most features contribute little to the data classification. Only a few features contribute most to the classification. Distribution of coefficient values has a nearly log-normal distribution. **(C)** Prediction accuracy of the GLM models trained with different numbers of most contributing features. Training set (Blue) consists of 2962 laser trails from 12 rats and prediction set (Red) consists 296 trails from a separate animal. Accuracy was calculated based on a 10-fold cross-validation. Classification accuracy for the test set increases fast with input numbers of features at the beginning and slowly reaches a top accuracy with dimension numbers around 120. Then accuracy starts to drop slowly all the way to around 86%. Shaded area indicate standard error margin. **(D)** Averaged noxious laser stimulation probability vs. post-stimulus time. Rates were calculated from the most accurate GLM in the previous step. The blue line and the red line represent control stimulation and noxious stimulation, respectively. Dash lines indicate the standard error margin. Significant increase of pain prediction probability appears in a time window of 1 s to 2 s after noxious stimulation, indicating a robust feature of laser-induced pain. Control group kept a low value for the entire period and does not show any increase after the stimulation onset.

After observing a consistent pattern among all noxious stimulation trials, we moved on to check which property contributed most to the differences between noxious and innoxious trials. We fitted a GLM with 12 animals (trials *n* = 2962). After model training, each property had a β-value indicating its contribution to the data classification result (Figure 3A). As shown in Figure 3B; most properties had little contribution to the classification while a small portion of the properties contributed the most. To check the contribution of each LFP feature to a accuracy classification, we trained 336 GLM classifiers with each LFP feature as input. All classifiers showed an accuracy between 70% and 75% (Figure 3A).

Since each property contributed differently to the overall classification result, we would like to check how many properties were needed to perform a good classification. Properties were sorted by absolute β-values from the initial GLM training. Several GLMs were trained with an increasing number of properties with the largest absolute β-value. Two-thousand nine-hundred and sixty-two trials from 12 animals were used to train the model. Ten-fold cross-validations were applied to each GLM to evaluate the model fitting. Two-hundred and ninty-six trials from another animal were used as the prediction dataset. As shown in Figure 3C, there was an increase in accuracy with the increase of dimension numbers at the beginning. When dimension numbers reached around 100, accuracy reached its peak around 89% then started to decrease and finally dropped to a plateau of 86%.

To test if the trained classifiers response uniquely to the noxious stimulation, we sliced the whole data set into 1-s data segments. We calculated the nociception probability of all segments using the GLM classifier calculated in the previous step with the highest prediction accuracy. Figure 3D shows the averaged nociception probability vs. post-stimulation time of all segments. Data shows an elevated nociception probability shortly (1–2 s) after the noxious stimulation onset, while innoxious group kept at a low prediction value all the time.

## Discussion

### LFP Characters of Laser-Induced Pain

In the present study, we provided evidence that oscillatory activities of four previously reported pain related areas including ACC, OFC, S1 and PAG could be used to indicate whether the animal has received noxious laser stimulation. Among 336 checked LFP features, 81 features showed statistical difference between control and noxious stimulation group (Figure 2). We trained 336 GLM classifiers with each individual LFP features and did not found any classifier could reach a training accuracy higher than 75% (Figure 3A). Our results showed that even with the most statistically significant LFP feature, we could not build a perfectly reliable classifier. Adopting the combined information from all four recorded brain areas could improve the GLM classification rate greatly (Figure 3C). This is in line with the idea that a “pain center” may not exist (Ploner et al., 2016). Pain is a phenomenon that actively integrate multiple brain areas (Bastuji et al., 2016). Without a consolidated view of each dependency, each component only forms a small piece of the whole picture. Even with the most statistically significant LFP feature, we could not build a reliable GLM classifier to tell if the animal received noxious stimulation (Figure 3C).

In order to check if we found the robust features indicating the onset of noxious laser stimulation, we moved on to further tests. Among all trained GLMs, the most accurate GLM was used to perform a window-by-window evaluation of the pain score through the whole recording sessions. Figure 3D shows the averaged pain prediction rate with incremental post-stimulus time. Innoxious group remained at a low prediction pain probability (<5%) while noxious group exhibited a peak value of about 58% probability in 1 s to 2 s window right after the stimulation onset. The prediction score was lower here because of the slicing windows around laser stimulation were not strictly aligned to the onset time. Noxious group exhibited a higher prediction level over innoxious group across the whole period, which might be caused by two reasons: (1) noxious laser and innoxious laser were performed in two separated sessions during the recording. Repeated high level stimulation caused a certain level of expectation, fear and potential hyperalgesia; which may explain the long-term rise of prediction level. (2) Movement-related neural activities/artifacts. Compared to the innoxious laser stimulation, the animal given the noxious laser tended to be hyperactive thus moved around more. We can only resolve this issue by introducing a movement control group.

We inspected δ, θ, α, β, γ, ε band power in the recorded brain areas shortly after the laser onset (Table 1). We found that decreased β band power in the ACC, OFC and S1 was most distinguishable between noxious group and innoxious group. It has been reported previously that β band power decreases during phasic pain (Hauck et al., 2015; LeBlanc et al., 2016). Another prominent change we observed was γ and ε band power increase in the ACC, OFC and S1. γ oscillation is an indicator of local computation (Buzsáki and Schomburg, 2015). Increased γ oscillation in S1 has been reported during phasic pain (Hauck et al., 2007; Gross et al., 2012).

**Table 1:** Top 20 most pain-contributing features in generalized linear model (GLM).

	Feature	Coefficient
1	OFC:ε	0.59389
2	ACC:β	−0.52565
3	S1:β	−0.51113
4	OFC:δ	0.50112
5	S1:ε	0.46285
6	PAG:δ	0.42436
7	S1:θ	0.38355
8	S1−S1:α-ε	−0.35997
9	OFC:γ	0.31415
10	ACC:ε	0.31415
11	OFC:β	−0.31013
12	OFC:θ	−0.30439
13	ACC−OFC:θ-ε	−0.28139
14	OFC−OFC:δ-α	−0.27765
15	PAG:ε	0.23902
16	S1−OFC:δ-δ	−0.23640
17	OFC−ACC:δ-β	0.23201
18	S1:δ	0.23035
19	OFC−ACC:α-γ	−0.22717
20	PAG−S1:α-ε	−0.22656

*Top 20 most pain-contributing features are listed in a descending order. Corresponding β values are listed in the last column. A positive β value means that an increase in the corresponding feature correlates to pain and vice versa*.

### Movement Artifact

Electrophysiology technique records voltage difference between electrodes and reference ground ranging from several hundreds of microvolts to several millivolts. Muscle contraction will generate movement artifact in the electrophysiology data. Pain related electrophysiology studies are affected by this problem more directly. In most pain research diagrams in animals, a certain behavior will be selected as an indicator of pain. It is necessary to remove the movement artifact before going into any further analysis.

In the present study, efforts were made to improve the noise removal during data pre-processing. This task started from careful inspection of LFPs trial by trial to remove any bad trials. The analyzing window was also chosen carefully, i.e., the LFP period between 500 ms and 1500 ms was used as the analyzing window. By doing this, we avoided the spectrum contamination from laser evoked potentials (LEPs) or from potentials related with movement such as walking and licking (Figure 1). Then ICA was used to remove all suspicious components (correlated noises). ICA is a powerful tool to identify different sources by their spatial distribution (Schomburg et al., 2014). In our experiment set up, we had the luxury to identify the movement noise better for the large spatial extent of electrodes. Noise was more “synchronized” in time. So it was identified as an independent component and thus removed (Supplementary Figure S2). But ICA could only remove the synchronized components from signal. If there were phase differences between recording channels, we could not remove the noise by this method. To get a better evaluation on our model, a separate movement control group should be added to consolidate the results.

### Neural Oscillation as a Potential Indicator of Pain

Event-related potentials are widely applied in analyzing laser-induced nociception signals (Iannetti et al., 2008; Bastuji et al., 2016). Event-related potentials are low in amplitude and usually need LFPs to be averaged over different trials. Compared to event-related potentials, neural oscillations are more prominent in amplitude in the LFP signals.

Neural oscillation could be easily quantified online with a filter-based algorithm. Field potential parameters have been used to build real-time closed-loop systems for a long time (Berényi et al., 2012; Priori et al., 2013; Krook-Magnuson et al., 2015). Such a system usually uses a distinct oscillation feature to control its outputs. Closed loop cancellation of ripples is a good example of such a device. Hippocampal ripple is a distinct oscillation feature and closely related to episodic memory (Buzsáki, 2015). A filter-based detector could easily quantify the amplitude of ripples. Then a threshold could be set to decide when to give stimulation. A classifier could use those neural oscillation measures to tell if the subject is in pain or not. Based on our present study, we could put the top pain-contributing parameters into an online detecting device and give out a real-time pain-score.

In this work, we studied the laser noxious stimulation-related LFP changes in four pain-related brain areas. Our results showed the possibility to use neural oscillation features to predict pain. But it is still unclear what aspects of the noxious stimuli drove the LFP signatures we found. Multiple regions and different neural processes may have involved in nociceptive signal processing. With higher density of recording electrodes and a broader coverage of recording sites, more pain-related activities could be monitored to get a better prediction result as well as a better source localization. The analysis framework used in this work could be further adopted to analyze electroencephalograph (EEG) data. In clinical practice, there is a great need of objective pain evaluation systems. Till now, the visual analog scale (VAS) is still being widely used (Hawker et al., 2011; Gagliese and Melzack, 2016; Spire et al., 2017). The VAS is a subjective pain reporting tool. It is hard to interpret under the same standard across different subjects. An objective pain evaluating method like the one we proposed in the present study might help doctors to objectively evaluate whether a patient suffered from pain and thus guide clinical practice accordingly. Objective pain evaluating system is also critical to animal researches. When evaluating pain in an animal, the only way till now is through its external behaviors (Capone and Aloisi, 2004; Pickering et al., 2006). An objective pain scoring method could be very valuable to better understand experiment results.

### Limitations

In this work, we have only recorded from four pain-related regions. More areas should be recorded when recording technique is available. When analyzing coupling properties, we did not take propagation delay into consideration to reduce computation complexity.

We only applied a simple laser pain test in the experiment. More stimulation types should be included in the further experiments to narrow down the candidates for pain-indication.

For future studies, stimulation should be introduced to check the physiology function of each correlated oscillation.

## Author Contributions

XL designed the experiment. XL and ZZ conducted the experiment. ZZ designed the data analysis routine and wrote corresponding scripts. JM and SC pre-processed the data with ICA. MY provided help with experiment idea. HG and YW mentored the experiment.

## Conflict of Interest Statement

The authors declare that the research was conducted in the absence of any commercial or financial relationships that could be construed as a potential conflict of interest.

## Footnotes

^1^http://statweb.stanford.edu/~wavelab/

## Supplementary Material

FIGURE S1 Local field potential (LFP) features of laser-stimulation trials in 2-D space. By adopting t-distributed stochastic neighbor embedding (t-SNE), high dimension LFP properties were casted into a low dimension space with maximal information entropy reserved. Trials with similar LFP properties had a smaller distance in between the corresponding data points in 2D space. To avoid overcrowding, comparisons were made in four sub-groups (Control vs. Pain, Pre- vs. Post-stimulation). The overlapped grayscale images were mean pain scores (laser trials with foot-lifting behavior). Data points were collected by all valid trials from 13 animals. After normalization, data points were not clustered into sub-groups by animal differences.

FIGURE S2 LFP denoising with independent component analysis (ICA). **(A)** Unprocessed LFP traces of four recorded areas (red: anterior cingulate cortex (ACC), purple: orbitofrontal cortex (OFC), green: primary somatosensory cortex (S1), blue: periaqueductal gray (PAG)). The vertical black line indicates the stimulus onset. Note the large movement artifact shows on S1 after stimulation onset. **(B)** ICA components of raw LFPs. Large and slow ICAs correlated to movement are candidates for movement artifact components. **(C)** LFP traces after noise elimination by ICA. **(D)** ICA coefficients. ICA components with uniform distribution across different recording channels are prone to be noise (like component 2).

FIGURE S3 Histology confirmation of electrode location. Red arrows point to burn marks of the electrodes in the ACC and OFC **(A)**, S1 **(B)** and PAG **(C)**. Blue traces are overlapped with rat atlas (Paxinos and Watson, 2009).
