# Supplementary figures and images for: Extracting Neural Oscillation Signatures of Laser-Induced Nociception in Pain-Related Regions in Rats
Source: Front Neural Circuits. 2017 Oct 9;11:71. doi: 10.3389/fncir.2017.00071 (PMC5640783; doi:10.3389/fncir.2017.00071)

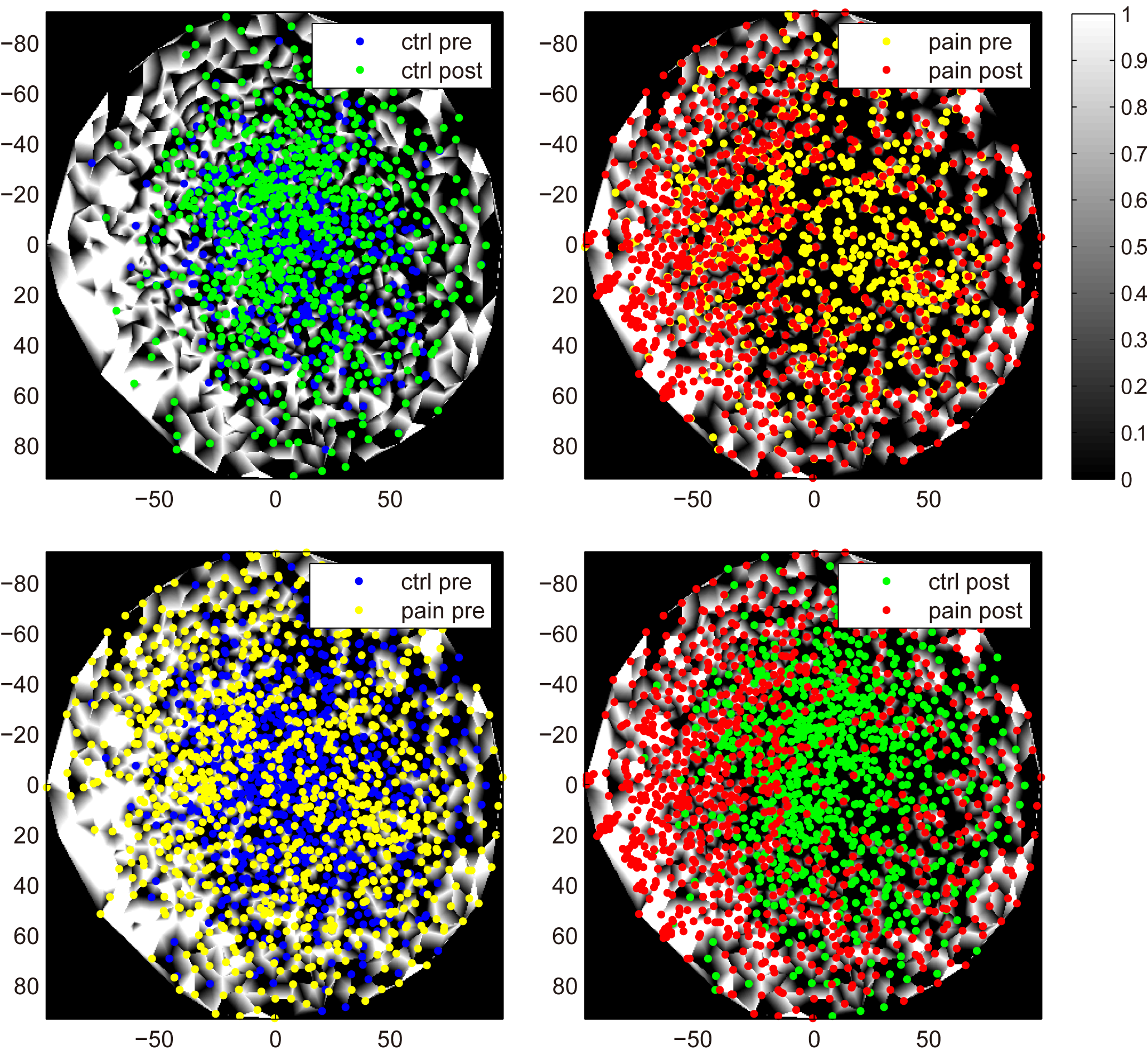

Supplement: FIGURE S1 — Local field potential (LFP) features of laser-stimulation trials in 2-D space. By adopting t-distributed stochastic neighbor embedding (t-SNE), high dimension LFP properties were casted into a low dimension space with maximal information entropy reserved. Trials with similar LFP properties had a smaller distance in between the corresponding data points in 2D space. To avoid overcrowding, comparisons were made in four sub-groups (Control vs. Pain, Pre- vs. Post-stimulation). The overlapped grayscale images were mean pain scores (laser trials with foot-lifting behavior). Data points were collected by all valid trials from 13 animals. After normalization, data points were not clustered into sub-groups by animal differences. [file Image_1.tif]

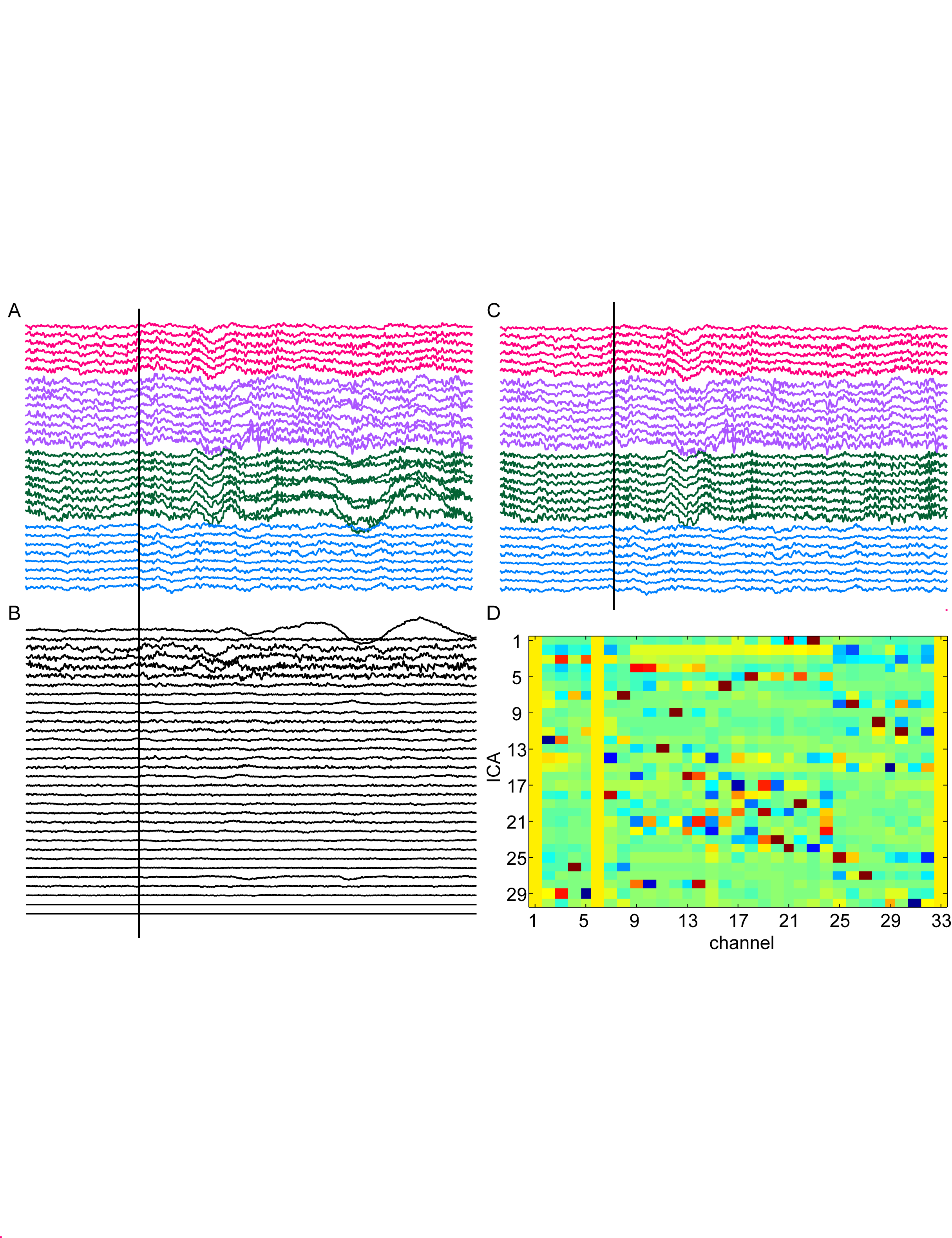

Supplement: FIGURE S2 — LFP denoising with independent component analysis (ICA). (A) Unprocessed LFP traces of four recorded areas (red: anterior cingulate cortex (ACC), purple: orbitofrontal cortex (OFC), green: primary somatosensory cortex (S1), blue: periaqueductal gray (PAG)). The vertical black line indicates the stimulus onset. Note the large movement artifact shows on S1 after stimulation onset. (B) ICA components of raw LFPs. Large and slow ICAs correlated to movement are candidates for movement artifact components. (C) LFP traces after noise elimination by ICA. (D) ICA coefficients. ICA components with uniform distribution across different recording channels are prone to be noise (like component 2). [file Image_2.tif]

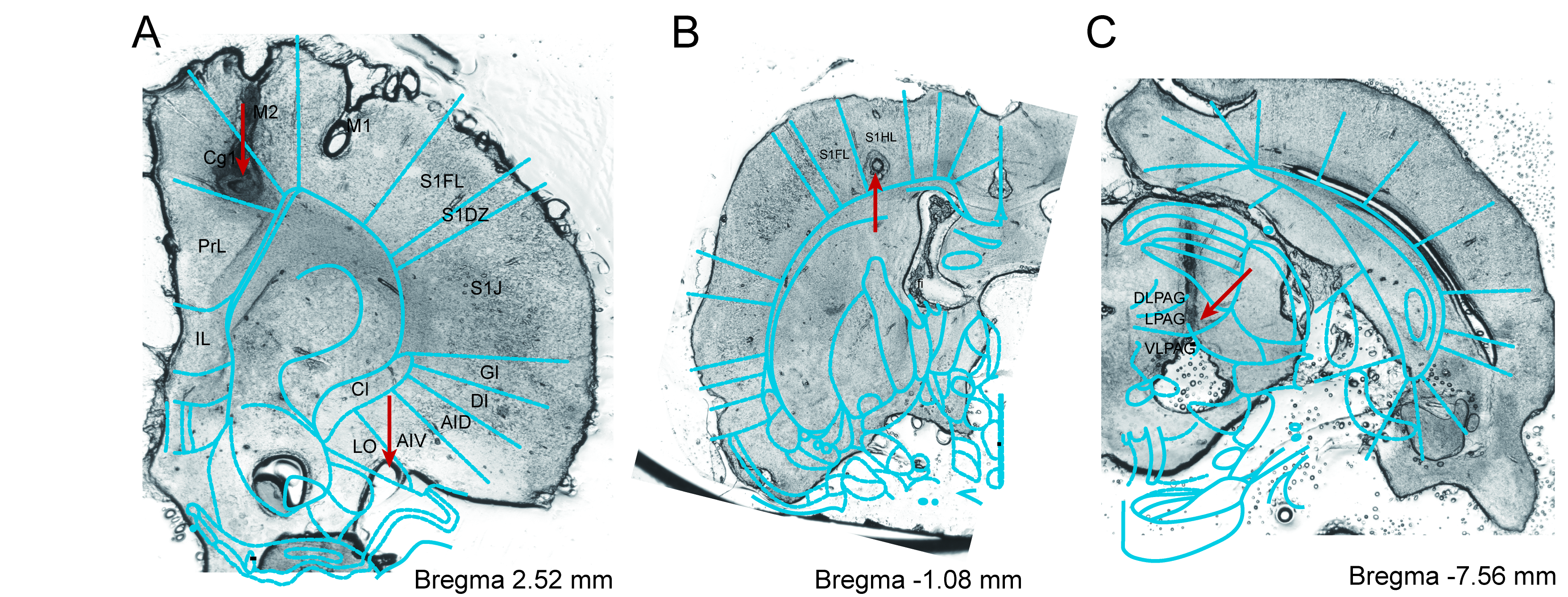

Supplement: FIGURE S3 — Histology confirmation of electrode location. Red arrows point to burn marks of the electrodes in the ACC and OFC (A), S1 (B) and PAG (C). Blue traces are overlapped with rat atlas (Paxinos and Watson, 2009). [file Image_3.tif]
